# Diabetic Uterine Environment Leads to Disorders in Metabolism of Offspring

**DOI:** 10.3389/fcell.2021.706879

**Published:** 2021-07-26

**Authors:** Ming-Zhe Dong, Qian-Nan Li, Li-Hua Fan, Li Li, Wei Shen, Zhen-Bo Wang, Qing-Yuan Sun

**Affiliations:** ^1^Institute of Reproductive Science, College of Life Sciences, Qingdao Agricultural University, Qingdao, China; ^2^State Key Laboratory of Stem Cell and Reproductive Biology, Institute of Zoology, Chinese Academy of Sciences, Beijing, China; ^3^University of Chinese Academy of Sciences, Beijing, China; ^4^Fertility Preservation Lab, Reproductive Medicine Center, Guangdong Second Provincial General Hospital, Guangzhou, China

**Keywords:** diabetes, uterus, metabolism, epigenetic inheritance, obese

## Abstract

**Aims:**

Research evidence indicates that epigenetic modifications of gametes in obese or diabetic parents may contribute to metabolic disorders in offspring. In the present study, we sought to address the effect of diabetic uterine environment on the offspring metabolism.

**Methods:**

Type 2 diabetes mouse model was induced by high-fat diet combined with streptozotocin (STZ) administration. We maintained other effect factors constant and changed uterine environment by zygote transfers, and then determined and compared the offspring numbers, symptoms, body weight trajectories, and metabolism indices from different groups.

**Result:**

We found that maternal type 2 diabetes mice had lower fertility and a higher dystocia rate, accompanying the increased risk of offspring malformations and death. Compared to only a pre-gestational exposure to hyperglycemia, exposure to hyperglycemia both pre- and during pregnancy resulted in offspring growth restriction and impaired metabolism in adulthood. But there was no significant difference between a pre-gestational exposure group and a no exposure group. The deleterious effects, no matter bodyweight or glucose tolerance, could be rescued by transferring the embryos from diabetic mothers into normal uterine environment.

**Conclusion:**

Our data demonstrate that uterine environment of maternal diabetes makes critical impact on the offspring health.

## Introduction

Diabetes is one of the largest global health emergencies of twenty-first century (Overland et al., 2014; Karuranga et al., 2019). The prevalence rate for obesity and type 2 diabetes has increased globally over recent decades at a pace. Maternal diabetes is a pathologic state that increases the incidence of complications in both the mother and the fetus (Vrachnis et al., 2012), for example, low quality oocytes (Zhang et al., 2013), increased chances of miscarriage (Mills et al., 1988) and congenital malformations of offspring (Vitoratos et al., 2010). Moreover, diabetic persons are considered to have significant positive relation for formation of hypercholesterolemia (Chowdhury and Nessa, 2019) and high LDL cholesterol (Jayakumari et al., 2020). Previous study has demonstrated that epigenetic inheritance via gametes by itself could increase an offspring’s susceptibility to develop to obesity and type 2 diabetes (Huypens et al., 2016). Specifically, we have found that paternal prediabetes increases the susceptibility to diabetes in offspring through gametic epigenetic alterations (Wei et al., 2014). The epigenetic profiling of pancreatic islets was changed in offspring depending on paternal prediabetes (Wei et al., 2014). Another study shows that sperm tsRNAs represent a paternal epigenetic factor that may mediate intergenerational inheritance of diet-induced metabolic disorders (Chen et al., 2016). Injection of sperm tsRNA fractions from high fat diet (HFD)-fed males into normal zygotes generated metabolic disorders in the F1 offspring (Chen et al., 2016). On the other hand, other studies show that gestational diabetes may have effects on offspring (Fetita et al., 2006; Sasson et al., 2015). An exposure to HFD only during gestation resulted in fetal growth restriction and decreased placental weight (Fetita et al., 2006; Sasson et al., 2015). We still know little about the effects of maternal diabetes on the growth and metabolism of offspring and how to change this kind of susceptibility to prevent this destructive cycle of metabolic dysfunction through generations.

Although the adverse effects of diabetic mothers on offspring may be owing to the epigenetic modifications in oocytes of diabetic mothers, the poor intrauterine environment may also play important roles. Previous studies have shown that offspring of gestational obese mothers or mothers fed a high-fat diet during gestation not only exhibited metabolic disorders but also impaired skeletal muscle development (Levin and Govek, 1998; Bayol et al., 2005; Huang et al., 2017). In our previous study, we showed that although embryo development was adversely affected by maternal diabetes, no evident imprinting abnormality was observed in oocytes from female offspring derived from a diabetic mother, and methylation in offspring’s oocytes is normal (Ge et al., 2013b). We further showed that the effects of maternal diabetes on imprinted genes might primarily be caused by the adverse uterus environment (Ge et al., 2013a). We do not clearly know which reason, uterus environment or gamete epigenetic inheritance, could be more important to increase offspring’s susceptibility to obesity or type 2 diabetes. To this end, we successfully generated a severe type 2 diabetic mouse model, and by zygote transfer demonstrated that uterine environment of maternal diabetes makes more impact on the offspring compared with epigenetic inheritance via gametes.

## Materials and Methods

Male and female C57BL/6J mice provided by SPF (Beijing) Biotechnology Co. Ltd. were housed in ventilated cages on a 12-h light/12-h dark cycle at a constant temperature (23 ± 1°C) and under controlled humidity (60 ± 5%). Mice were *ad libitum* fed water and food. All mouse experiments were approved by the Ethics Committee of the Institute of Zoology, Chinese Academy of Sciences.

### Diabetic Mouse Model

We generated a type 2 diabetes mouse model according to previous studies, with slight changes (Wei et al., 2014). In brief, 3 weeks old C57BL/6J mice, five per cage, were randomly divided into two groups and fed with either a HFD (D12492; Research Diet, New Brunswick, NJ, United States) or a normal standard chow (NC, SPF Rodent Growth and Breeding Feed, BEIJING KEAO XIELI FEED CO., LTD) until 20 weeks of age. During 12–15 weeks old, mice fed with HFD diet received intraperitoneal injection of a sub-diabetogenic dose of STZ (100 mg/kg body weight, S0130, Sigma) once a week, a total of 4 times, and kept on the same diet until 20 weeks old. The fasting plasma glucose was measured after overnight fasting and the casual plasma glucose was measured at any point in time by a glucometer (Accu-CHEK active; Roche Diagnostic). The measurement was executed every week between 17 and 20 weeks of age after STZ injection. Mice with a casual plasma glucose ≥ 200 mg/dl (11.1 mmol/l) or a fasting plasma glucose ≥ 126 mg/dl (7.0 mmol/l) were considered as having diabetes.

### Experimental Design

**FIGURE 1:**
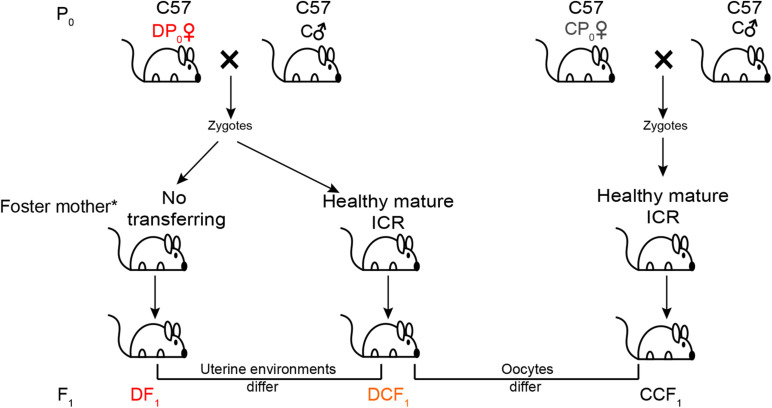
Experimental design. Maternal C57BL/6J mice (P_0_) were at random allocated to induce or not induce diabetes. The diabetic mice (DP_0_) and non-diabetic mice (CP_0_) were mated with normal C57BL/6J males (C♂) and their zygotes were collected and transferred into the oviduct of healthy mature ICR foster mother. For the other part of successfully mated DP_0_ mice, their zygotes were not transferred and incubated in the original diabetic uterus of DP_0_. Newborn offspring (F_1_) of these three group were all fed by ICR foster mother. All offspring were weaned at 3 weeks of age and placed on a normal diet. *All offspring were breast fed by ICR mothers.

To test the impact of uterine environment on metabolism of offspring, female type 2 diabetes mice (hereafter called DP_0_) about 21 weeks old were mated with normal C57BL/6J males for generating offspring (DCF_1_ and DF_1_) from different uterine environment by transferring the zygotes into healthy mature foster ICR (Institute of Cancer Research) mothers or no transferring (Figure 1). On the other hand, to test whether the metabolism of offspring could be affected by gamete epigenetic inheritance or not, the zygotes of female normal diet control mice (hereafter called CP_0_) as same age as DP_0_ mated with normal C57BL/6J males were also transferred into healthy foster ICR mothers to generate offspring (CCF_1_) which were compared with DCF_1_ (Figure 1). Each foster mother would receive 6–12 zygotes. The number of litters per group was not less than 4. All offspring were breast fed by ICR mothers and subjected to normal standard chow after weaning. Each ICR mothers feed pups no more than 6 to ensure enough milk. We also designed an untreated group, in which the normal maternal C57BL/6J mice were mated with normal C57BL/6J males with no zygotes transfer to analyze the reproductive phenotype as control. These offspring of CP_0_ is named CF_1_.

### Zygote Collection and Offspring Production

Zygote collection and transfer were conducted following standardized procedures described in the Manipulating the Mouse Embryos. In brief, C57BL/6J females (HFD and NC) in heat were mated with normal C57BL/6J males between 20 and 22 weeks of age, and zygotes were collected from a part of the successfully mated female mice. The collected zygotes of each female mouse were analyzed and subsequently used for oviduct transfer into ICR recipient mother on normal diet. Each foster mother would receive 6–17 zygotes which were divided into two parts and transferred into the bilateral oviducts. The birth rate was the ratio of birth number to the number of embryos transferred. F_1_ of the two groups (DCF_1_ and CCF_1_) were all fed by ICR mice during lactation. DF_1_ mice were generated by another part of the successfully mated female HFD mice and fed by ICR mice during lactation in an identical fashion.

### Dystocia Rate and Cesarean Section

If the pregnant female mouse could not attempt the vaginal delivery at day 21 of pregnancy, it was classified as dystocia and recorded. The dystocia rate was the ratio of dystocia number to the number of all pregnancy in each group. Then cesarean section was conducted right after cervical dislocation by hysterectomy, and pups were quickly wiped clean and placed on a warming plate at 37°C. Before transferring to a foster mother, the survival number and abnormal number of each litter were recorded. For the vaginal delivery pups, the survival number and abnormal number of each litter were recorded at the first day they were born. The survival rate of each group was worked out by dividing the survival number by the total birth number. However, the abnormal rate of each group was worked out by dividing the abnormal number after weaning by the total birth number, because some malformations in eye was diagnosed until weaning. A minimum of 4 litters were used in each group.

### Body Weight Measurement

The body weights of maternal mice were measured every week between 7 and 21 weeks of age. The newborns’ weights were measured at the 1 day of age. Then, we weighed the offspring every week from 3 to 15 weeks of age.

### ITT and GTT

ITT (insulin tolerance test) and GTT (glucose tolerance test) were performed as previously described (Wei et al., 2014) and the offspring of each group used for the glucose tolerance were from different litters. In brief, ITT was conducted after a fasting period of 2 h. Mice received 1 unit of insulin (I-5500, Sigma) per kilogram of body weight by intraperitoneal injection. The concentration of insulin solution used for injection was 0.05 U/ml. Blood glucose levels from tail blood were determined at 0, 15, 30, 60, and 120 min after application of the insulin by a glucometer. GTT was conducted after an overnight fasting period of 13 ± 1 h. Mice received 2 g of glucose per kilogram of body weight by intraperitoneal injection. Glucose was dissolved in water to 10% (mass-to-volume) concentration and used to inject. Blood glucose levels from tail blood were determined at 0, 15, 30, 60, and 120 min after application of the glucose challenge by the same glucometer. Offspring of different litters (no less than 4 in each group) were randomly selected and analyzed for the test.

### Serum Biomarkers Test

Serum was isolated from blood sampled by eyeball extirpating and stored at −80°C until detection. HuNan FengRui Biotechnology Co., Ltd. was employed for biochemical detection of serum. The tested items include the total cholesterol (TC), the triglyceride (TG), high-density lipoprotein cholesterol (HDL) and low-density lipoprotein cholesterol (LDL).

### Statistical Analysis

All analyses were performed using GraphPad Prism 5 software. Results were expressed as mean ± S.E.M. For all comparisons that involved multiple time points, a two-way repeated measurement ANOVA, *post hoc* Bonferroni multiple-comparisons test was used to assign *P*-values by comparing to each other in the F1 generation. In the P0 generation, CP0 ♀served as control group. For measurements at single time points, a one way ANOVA was used. To assess differences in blood glucose of P0 generation, *post hoc* Bonferroni multiple-comparisons test was applied to assign *P*-values vs. the control group. To assess differences in body weight distribution in F1 cohorts, a Kruskal-Wallis test, using Dunn’s multiple-comparisons test, was applied as a non-parametric test to assign *P*-values. The D’Agostino and Pearson omnibus normality test was applied to assess the survival rate and abnormality rate of F1 in each litter. A two-tailed Student’s t test was performed to compare two different groups, and an unpaired test was performed on non-parametric data *P*< 0.05 was considered statistically significant.

## Results

### Establishment of the Diabetes Mouse Model

**FIGURE 2:**
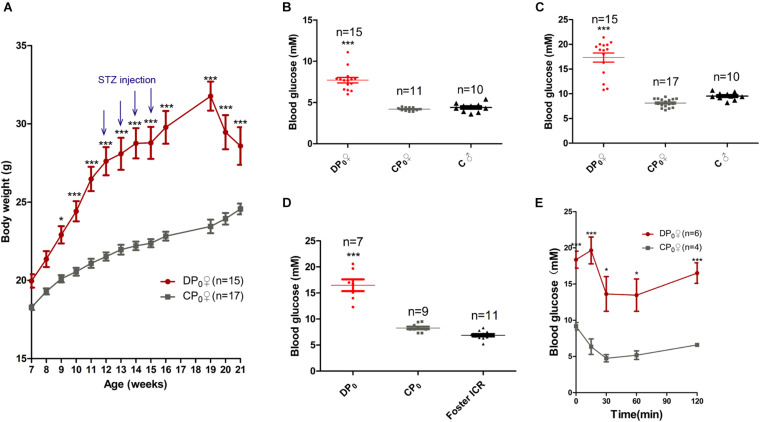
Maternal (P_0_) metabolic phenotype. **(A)** Body weight gain from 7 to 21 weeks. **(B)** Fasting plasma glucose. **(C)** Casual plasma glucose. **(D)** Gestational plasma glucose. **(E)** ITT at 21 weeks of age. Data are expressed as mean ± S.E.M.; *P < 0.05, ***P < 0.001 vs. CP_0_ control. A two-way repeated measurement ANOVA in **(A,E)** and a one way ANOVA in **(B–D)**, *post hoc* Bonferroni multiple-comparisons test was used to assign *P*-values. *P*-values for significance between groups in repeated measure analysis are shown.

To explore which has more effects on the offspring health of diabetes, uterine environment or oocytes, we generated type 2 diabetes mouse model by feeding high fat diet combined with STZ, a diabetagen which is especially toxic to pancreatic islet insulin-producing β-cells, injection at a low dose. The body weight of high fat diet type 2 diabetes mice (DP_0_) was significantly higher than that of normal diet control mice (CP_0_), when measured between 7 and 21 weeks of age. However, the body weight of DP_0_ was increased before 19 weeks of age, and subsequently decreased in 3 weeks after STZ injection (Figure 2A). According the diagnostic criteria for diabetes mentioned in the materials and methods, we picked 34 mice which successfully developed diabetes from 50 suffering treatment for the following research. Sixteen mice which did not develop diabetes were excluded from the study. The fasting plasma glucose and the casual plasma glucose during pre-gestational period, as well as the casual plasma glucose during gestational period of DP_0_ were all significantly higher than in CP_0_ mice (Figures 2B–D, 7.61 ± 0.41 vs. 4.2 ± 0.06, 17.33 ± 0.9 vs. 8.13 ± 0.2, 14.97 ± 1.25 vs. 8.29 ± 0.25, respectively, *P* < 0.001). Next, we analyzed insulin resistance of the female mice in both DP_0_ and CP_0_ when they were 21 weeks of age. The mice of DP_0_ showed decreased insulin sensitivity compared with controls (Figure 2E). On the other hand, because the plasma glucose in GTT was too high to be measured, data of GTT was not shown.

### Female Diabetic Mice Had Reduced Fertility, Accompanying the Increased Risk of Offspring Malformations and Death, Which Could Be Rescued by Zygote Transfer

**FIGURE 3:**
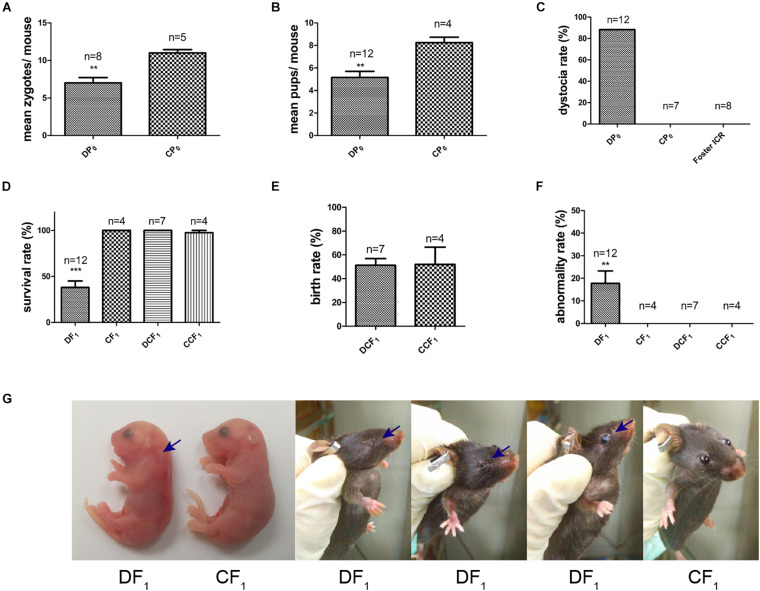
Maternal reproductive phenotypes and offspring symptoms. **(A)** The number of zygotes per mouse. **(B)** The number of pups per mouse. **(C)** The maternal dystocia rate. **(D)** Survival rate of F_1_ in each group. **(E)** Birth rate of DCF1 and CCF_1_. **(F)** Abnormality rate of F_1_ in each group. **(G)** Congenital malformations of DF_1_ compared to CF_1_. Blue arrows show abnormal regions. Data are expressed as mean ± S.E.M.; **P < 0.01, ***P < 0.001 vs. CP_0_ or CF_1_ control. A two-tailed Student’s *t*-test was performed in **(A,B,E)**. The D’Agostino and Pearson omnibus normality test was applied in **(D,F)**. *P*-values for significance between groups in repeated measure analysis are shown.

Female mice, DP_0_ or CP_0_, were mated with normal diet control male mice in which the fasting and casual plasma glucose were both normal (Figures 2B,C). The natural ovulation of per female mouse was counted when a vaginal plug was observed next morning. The number of natural ovulation was significantly lower in the diabetic group than control group by 20 weeks of age (Figure 3A, 7 ± 0.68 vs. 11 ± 0.4, *P*< 0.01). Associated with the decrease in natural ovulation, DP_0_ female mice become significantly low reproductive, with the offspring number of DP_0_ female mice decreased about 1.6-fold compared with the CP_0_ group (Figure 3B, 5.17 ± 0.51 vs. 8.25 ± 0.41, *P*< 0.01). The dystocia rate of DP_0_ female mice also was very high compared with CP_0_ female mice (Figure 3C, 88 and 0%, respectively).

Meanwhile, the survival rate of offspring, DF_1_ exposed to diabetic uterine, was decreased to only 38% compared to CF_1_ (Figure 3D). However, we did not observe differences in survival rate and birth rate between the two transfer groups, DCF_1_ and CCF_1_, when zygotes of DP_0_ mice or CP_0_ mice were collected and transferred into control ICR recipients (Figures 3D,E). The offspring exposed to maternal diabetes during gestation also manifested severe congenital malformation vs. offspring of control, with the abnormality rate of 18% (Figure 3F) in DF_1_ group. The congenital malformation includes neck hypertrophy and eye diseases (anophthalmia, microphthalmia, and white eye disease) (Figure 3G). There was also no congenital malformation being detected in the offspring when the zygotes were transferred into normal uterine environment (Figure 3F).

### Diabetic Uterine Environment Caused Lower Birth Weight and Growth Restriction After Birth

**FIGURE 4:**
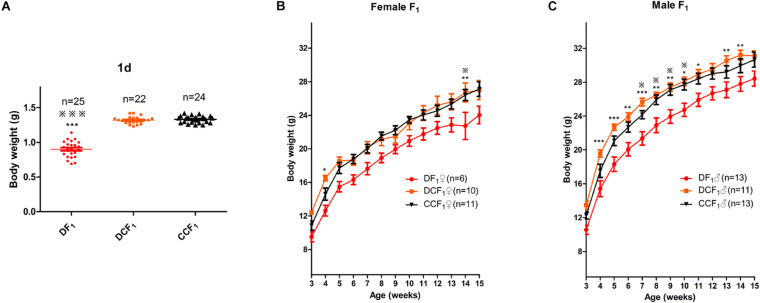
F_1_ body weight distributions and trajectories. **(A)** Scatter plots of the body weight distribution for F_1_ at 1 day of age. **(B,C)** The body weight trajectories of female F_1_ **(B)** and male F_1_ **(C)** mice between 3 and 15 weeks of age. Data are expressed as mean ± S.E.M.; *P < 0.05, **P < 0.01, ***P < 0.001 DF_1_ vs. DCF_1_. Statistical significance, DF_1_ vs. CCF_1_, is represented using 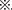. There is no statistical significance between DCF_1_ and CCF1. A one way ANOVA in **(A)** and a two-way repeated measurement ANOVA in **(B,C)**, *post hoc* Bonferroni multiple-comparisons test was used to assign *P*-values by comparing to each other. *P*-values for significance between groups in repeated measure analysis are shown.

To further explore the effect of diabetic uterine environment to the offspring, we analyzed the weight between DF_1_, DCF_1_, and CCF_1_. As shown in Figure 4A, DF_1_ mice had lower birth weight than DCF_1_ (0.90 ± 0.02 vs. 1.32 ± 0.01, *P* < 0.001), while there was no significant difference between DCF_1_ and CCF_1_ (1.32 ± 0.01 vs. 1.33 ± 0.01, *P* > 0.05). Then the pups of all three groups were fed by ICR mice until to weaning. After weaning, the three groups were all fed normal standard chow and the body weight was recorded from 3 to 15 weeks old. Female offspring from diabetic mother and diabetic uterine environment, DF_1_♀, demonstrated growth restriction compared with DCF_1_♀and CCF_1_♀especially at 4 and 14 weeks of age, showing significantly different (Figure 4B). However, there was no significant difference in body weight between DCF_1_♀and CCF_1_♀(Figure 4B). The distributions of body weights in male F_1_ cohorts of the three groups demonstrated the corresponding curve. What was different was that the growth restriction of DF_1_♂ was more significant compared to DCF_1_♂ and CCF_1_♂ from 3 to 15 weeks of age (Figure 4C). However, there was no significant difference in body weight between DCF_1_♀and CCF_1_♀ (Figure 4C).

### DF_1_ Exhibited Impaired Metabolism, While the Metabolism of DCF_1_ Was Normal

**FIGURE 5:**
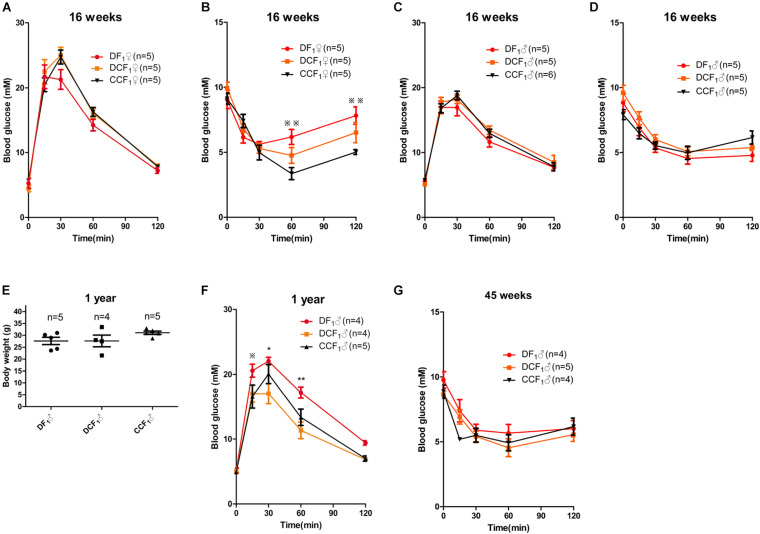
F_1_ metabolic phenotype. **(A,C)** Blood glucose concentrations during GTT in female **(A)** and male **(C)** F_1_ mice at 0, 15, 30, 60, and 120 min after intraperitoneal glucose injection when they were 16 weeks of age. **(B,D)** Blood glucose concentrations during ITT in female **(B)** and male **(D)** F_1_ mice at 0, 15, 30, 60, and 120 min after intraperitoneal insulin injection when they were 16 weeks of age. **(E)** Scatter plots of the body weight distribution for male F_1_ at 1 year of age. **(F)** Blood glucose concentrations during GTT in male F_1_ mice at 0, 15, 30, 60, and 120 min after intraperitoneal glucose injection when they were 1 year of age. **(G)** Blood glucose concentrations during ITT in male F_1_ mice at 0, 15, 30, 60, and 120 min after intraperitoneal insulin injection when they were 45 weeks of age. Data are expressed as mean ± S.E.M.; *P < 0.05, **P < 0.01 DF_1_ vs. DCF_1_. Statistical significance, DF_1_ vs. CCF_1_, is represented using 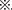 There is no statistical significance between DCF_1_ and CCF1. A two-way repeated measurement ANOVA in **(A–D,F)** and a one way ANOVA in **(E)**, *post hoc* Bonferroni multiple- comparisons test was used to assign *P*-values by comparing to each other. *P*-values for significance between groups in repeated measure analysis are shown.

To investigate whether the metabolism of offspring was affected by uterine environment or oocytes of maternal diabetes, we analyzed glucose tolerance and insulin tolerance in all the three F_1_ cohorts when they were 16 weeks old. As shown in Figure 5A, the blood glucose levels during an intraperitoneal glucose tolerance test (GTT) were not significantly different among the female mice of the three groups. But, DF1 females showed insulin resistance in the ITT when they were 16 weeks old (Figure 5B). To the three male F_1_ cohorts, there was no significant difference in either GTT or ITT when they were 16 weeks old (Figures 5C,D). We continued to feed the three male F_1_ cohorts by normal diet until 1 year of age, which was equivalent to around 42.5 years old of human. As shown in Figure 5E, there was no significant difference in the body weight of the three groups. Then, we analyzed glucose tolerance in these middle age mice. Interestingly, DF_1_ males displayed an impaired glucose tolerance vs. DCF_1_ or CCF_1_ at this life phase (Figure 5F). However, DCF_1_ displayed a normal glucose tolerance vs. CCF_1_ (Figure 5F). We also analyzed the insulin tolerance when they were 45 weeks of age, but there was no significant difference among the three groups (Figure 5G).

## Discussion

People with diabetes are at higher risk of developing a number of disabling and life-threatening health problems than people without diabetes (Overland et al., 2014), such as diabetic eye disease, diabetic nephropathy and diabetic foot disease (Abuaisha et al., 1998; Apelqvist et al., 2000; Calvo-Maroto et al., 2014). In particular, women with any type of diabetes are at risk of a number of complications during pregnancy, as high glucose levels can affect the development of the fetus (Overland et al., 2014). However, we did not clearly know which factor, the oocytes from diabetes or diabetic uterine environment, plays the crucial role in the development of their offspring.

Maternal diabetes may induce delayed oocyte maturation, abnormal cellular metabolism, mitochondrial dysfunction and meiotic defects (Ratchford et al., 2007; Wang et al., 2009, 2010; Cheng et al., 2011). It is well known that oocytes are deficient in their ability to use glucose as an energy substrate and require cumulus cells to provide products of glycolysis for their own development (Sugiura and Eppig, 2005). Maternal diabetes may indirectly impair oocyte competence by disrupting mitochondrial function in cumulus cells and their communications with the oocytes (Wang et al., 2010), which could have an impact on the provision of essential nutrients or information molecules. In our present study, we also demonstrated reduced natural ovulation (Figure 3A) and lower reproductive ability (Figure 3B). In addition, oocyte development and maturation are usually accompanied by the establishment of maternal imprints (Saitou et al., 2012). Some imprinted genes appear to be involved in lipid and energy metabolism (Haig, 2004), and even affect postnatal metabolism (Millership et al., 2019). Previously, we found that maternal diabetes not only causes the alterations of DNA methylation statuses of some imprinted genes in maternal oocytes (Ge et al., 2013b), but also led to the alterations of the methylation patterns and expressions of the imprinted genes in mid-gestational placental tissues (Ge et al., 2013a).

On the other hand, the adverse uterine environment of maternal diabetes could cause an increase in placenta weight and elevated glucose levels in both plasma and amniotic fluid (Laurino et al., 2019). In the diabetes, higher placental weights showed reduced placental efficiency and inability to transfer nutrients to the developing fetus (Volpato et al., 2015). In addition to the placental influence, the high rate of glucose in the amniotic fluid can prolonged the fetuses β-pancreatic cells stimulation, causing a pancreatic insulin depletion and hypoinsulinemia (Lopez-Soldado and Herrera, 2003). At the crucial period of epigenetic reprogramming, adverse uterine environment may influence the establishment and maintenance of methylation in mice. For example, Martino et al. reported that placental mRNA abundance for the folate receptor alpha (FOLR1) and DNA methyltransferases (DNMT1) were influenced with raised BMI (Martino et al., 2018). Although the mechanisms remain to be clarified, the alterations of the methylation patterns and expressions of the imprinted genes in mid-gestational placental tissues (Ge et al., 2013a) may influence the postnatal metabolism of offspring (Crespi, 2019).

In the present study, we generated the type 2 diabetes mouse model, which exhibited the symptoms of overweight, high level fasting plasma glucose and insulin resistance (Figures 2A,B,E). In addition, the casual plasma glucose and gestational plasma glucose were abnormally high as well (Figures 2C,D). To compare the effect of uterine environment and oocytes on offspring, we analyzed the symptoms of the offspring from diabetic uterine environment or not by changing the single variable factor. As shown in the results, DF1 mice, offspring from diabetic uterine environment, exhibited lower survival rate and higher malformation rate (Figures 3D,F) than DCF_1_ mice, under the premise of no difference in embryo transplantation (Figure 3E). The malformation included neck hypertrophy during fetus and eye disease after birth (Figure 3G), which caused by the adverse uterine environment. For the maternal diabetes, the number of natural ovulation and dystocia rate both suffered adverse effect (Figures 3A,C), just like human beings with gestational diabetes (Overland et al., 2014). It was worth mentioning that our diabetic model had a far higher level of dystocia rate than human beings, which may be related to the severity of maternal diabetes. Because the female diabetic mice in our research had caught severe diabetes when they were pregnant which could be judged by the loss of their weight and the high gestational plasma glucose (Figures 2A,D).

In addition, gestational diabetes mellitus (GDM) and obesity are both complications which occur during pregnancy and subsequently influence the development of offspring during fetal life and postnatal development (Yessoufou and Moutairou, 2011). In our analysis, DF_1_ showed low birth weight and growth restriction after birth (Figure 4A), yet there was no significant difference between DCF_1_ and CCF_1_ when they were subjected to a normal diet (Figure 4). However DF_1_ displayed catch-up growth and the difference of weight among the three groups was gone until 16 weeks old (Figures 4B,C). We also examined the total cholesterol (TC), high-density lipoprotein cholesterol (HDL), low-density lipoprotein cholesterol (LDL), and the triglyceride (TG) of parents and offspring. For DP_0_, they had elevated HDL and TC compared with CP_0_, which may be responsible for the decreased body weight (Tan, 1986) and the hypercholesterolemia, although there were no significant difference in LDL and TG (Supplementary Figure 1). However, the hypercholesterolemia had not been passed on to the next generation, because there is no significant difference among DF_1_, DCF_1_, and CCF_1_ when they were 16 weeks old. It is possible that there are other metabolic abnormalities such as impaired beta-cell function, energy expenditure, physical activity or behavior changes that characterize these offspring (Sasson et al., 2015). It is clear that the risk of becoming diabetic is greater for relatives of diabetics than for individuals of the same age and sex in the general population (Simpson, 1968). Besides genetic factors, the offspring’s risk of developing NIDDM is influenced by environmental factors, especially obesity (Pierce et al., 1995). Type 2 diabetes is one form of diabetes, in which the insulin resistance of patients is mostly caused or aggravated by obesity (Alberti and Zimmet, 1998). Interestingly, no matter maternal undernutrition (Schulz, 2010; Hales and Barker, 2013) or maternal hyper-nutrition, e.g., obesity and maternal diabetes (Lowe et al., 2018; Hod et al., 2019), the offspring has a greatly increased susceptibility to the development of type 2 diabetes. For the undernutrition model, the thrifty phenotype hypothesis was proposed to explain the effects of poor nutrition in fetal stage to subsequent metabolic syndrome (Hales and Barker, 2001). However, contextually with the dramatic spread of obesity and type2 diabetes, the prevalence of GDM has significantly increased over the last few years (Zhu and Zhang, 2016; Filardi et al., 2019). The prevalence of GDM has increased by more than 30% within one or two decades in a number of countries including developing countries, forming an emerging worldwide epidemic (Guariguata et al., 2014; Zhu and Zhang, 2016).

In our present study, the offspring from diabetic uterine environment showed impaired metabolism and was recovered by transfer into normal uterus (Figure 5). In addition, the metabolic phenotypes displayed difference between sexes. As shown in Figure 5, female DF1 developed metabolic disorder earlier than male, as male DF1 displayed an impaired glucose tolerance until 1 year of age. The cause of this different phenotypes between sexes remains to be determined. However, this demonstrated that the impact of uterine environment on offspring’s metabolism is more important than gamete alone. Although more and more studies currently tend to suggest that it is the oocytes (Luzzo et al., 2012; Han et al., 2018) or spermatozoa (Wei et al., 2014; Chen et al., 2016; Huypens et al., 2016) of diabetic parents contribute to impact on offspring and emphasize the importance of epigenetic inheritance to increase an offspring’s susceptibility to developing obesity and type 2 diabetes. All these studies tried to explain the phenomenon that the prevalence rates for obesity and type 2 diabetes have increased globally over recent decades at a pace by epigenetic inheritance via gametes. As meanwhile, the concern of many researchers, during the last decade, is to explore the physiopathology of the relationship between the health conditions of offspring born from pregnancy complicated with diabetes (Yessoufou and Moutairou, 2011). Offspring developing impaired glucose tolerance or metabolic abnormalities differ according to the level of hyperglycemia during gestation and the period of life studied (Fetita et al., 2006). These studies were carried out in humans (Evers et al., 2004; Atègbo et al., 2006; Grissa et al., 2007; Mitanchez, 2010) and animal models, mostly in rats (Simmons et al., 2001; Lopez-Soldado and Herrera, 2003). As the controversy on the two viewpoints, we first experimentally tested the effect of diabetic uterine environment and gamete by inducing a severe type 2 diabetes mouse model and fertilized egg transfer. As we predicted, uterine environment may play a critical role in increased offspring’s susceptibility to obesity or type 2 diabetes. This may contribute to understanding the current obesity and diabetes pandemic.

Overall, we successfully generated a severe type 2 diabetic mouse model to demonstrate that uterine environment of maternal diabetes may make a more significant impact on the offspring health compared to epigenetic inheritance via gametes.

## Data Availability Statement

The original contributions presented in the study are included in the article/Supplementary Material, further inquiries can be directed to the corresponding author/s.

## Ethics Statement

The animal study was reviewed and approved by the Ethics Committee of the Institute of Zoology, Chinese Academy of Sciences. Written informed consent was obtained from the owners for the participation of their animals in this study.

## Author Contributions

MZD carried out the analysis, designed the assay, generated mouse models, and wrote the manuscript. QNL, LHF, and LL participated in analyzing the data. ZBW and WS participated in revising the manuscript. QYS designed and revised the manuscript. All authors read and approved the final manuscript.

## Conflict of Interest

The authors declare that the research was conducted in the absence of any commercial or financial relationships that could be construed as a potential conflict of interest. The handling editor declared a past co-authorship with one of the author WS.

## Publisher’s Note

All claims expressed in this article are solely those of the authors and do not necessarily represent those of their affiliated organizations, or those of the publisher, the editors and the reviewers. Any product that may be evaluated in this article, or claim that may be made by its manufacturer, is not guaranteed or endorsed by the publisher.
