# Supplementary material for: Diabetic Uterine Environment Leads to Disorders in Metabolism of Offspring
Source: Front Cell Dev Biol. 2021 Jul 26;9:706879. doi: 10.3389/fcell.2021.706879 (PMC8350518; doi:10.3389/fcell.2021.706879)
Supplement: Supplementary file 1 [file Data_Sheet_1.DOCX]

**
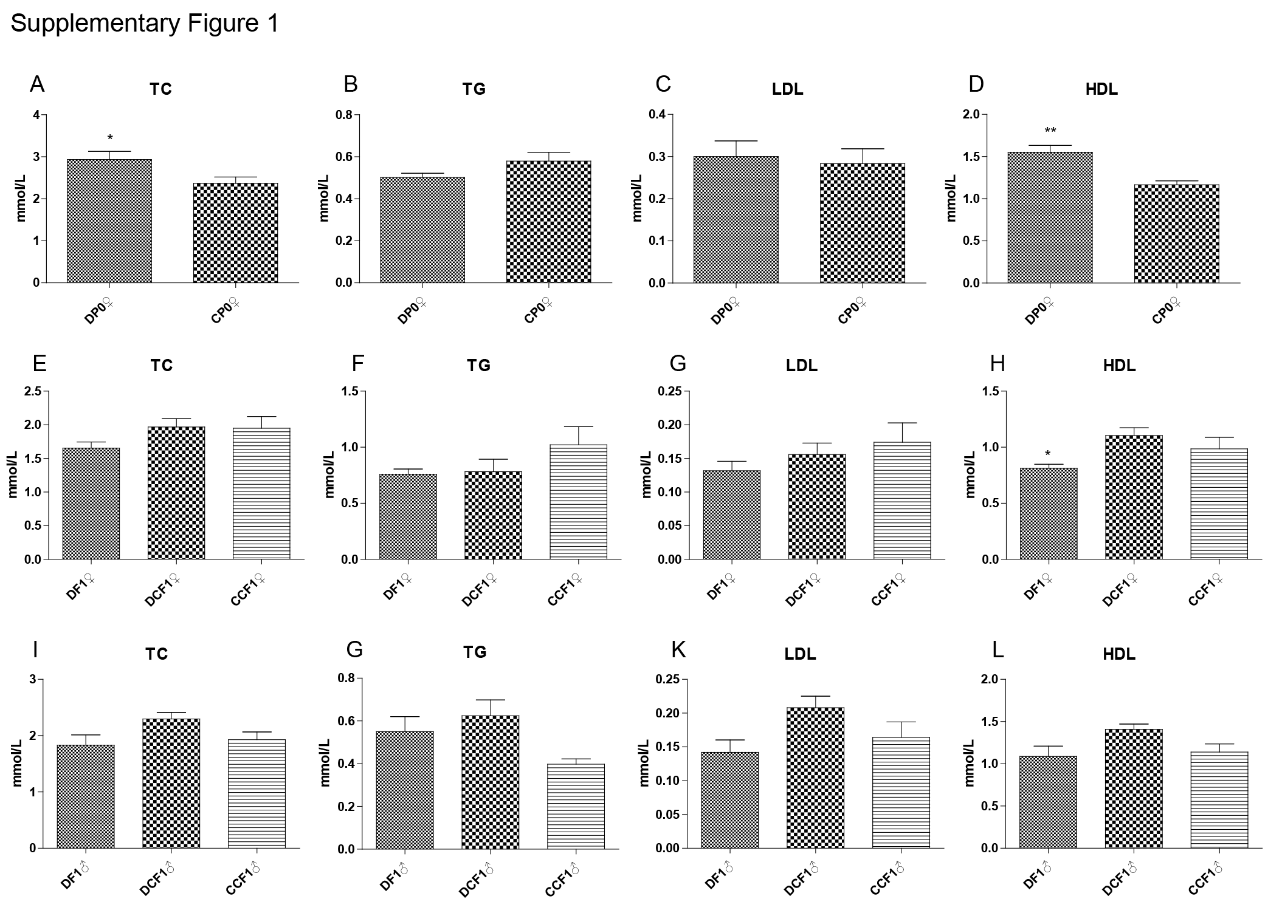
**

**Supplementary figure legend**

**Supplementary figure 1 |** Serum biomarkers parents and their offspring. (**A-D**) The total cholesterol (TC), the triglyceride (TG), high-density lipoprotein cholesterol (HDL) and low-density lipoprotein cholesterol (LDL) of mothers. The serum of mothers were isolated from blood when they were 28 weeks old (DP_0_♀, CP_0_♀: n=6 and 6, respectively). (**E-H**) The TC, TG, HDL and LDL of female F1. The serum were isolated from blood when they were 16 weeks old (DF_1_♀, DCF_1_♀ and CCF_1_♀: n=6, 5 and 5, respectively). (**I-L**) The TC, TG, HDL and LDL of male F_1_. The serum were isolated from blood when they were 16 weeks old (DF_1_♂, DCF_1_♂ and CCF_1_♂: n=5, 5 and 5, respectively). Data are expressed as mean ± S.E.M.; **P* < 0.05, **P < 0.01 DP_0_ versus CP_0_, **P* < 0.05 DF_1_ versus DCF_1_. There is no statistical significance between DCF_1_ and CCF_1_. A two-tailed Student’s *t* test was performed in **A-D**. A one way ANOVA, *post-hoc* Bonferroni multiple-comparisons test was used to assign P values by comparing to each other in **E-L**. *P* values for significance between groups in repeated measure analysis are shown.

**Supplementary methods**

**Serum biomarkers test**

Serum was isolated from blood sampled by eyeball extirpating and stored at -80℃ until detection. HuNan FengRui Biotechnology Co.,Ltd was employed for biochemical detection of serum. The tested items include the total cholesterol (TC), the triglyceride (TG), high-density lipoprotein cholesterol (HDL) and low-density lipoprotein cholesterol (LDL).
